# The neural bases of tactile vitality forms and their modulation by social context

**DOI:** 10.1038/s41598-021-87919-z

**Published:** 2021-04-27

**Authors:** G. Rizzolatti, A. D’Alessio, M. Marchi, G. Di Cesare

**Affiliations:** 1 Neuroscience Unit, Department of Medicine and Surgery, University of Parma, Parma, Italy; 2 Istituto di Neuroscienze, Consiglio Nazionale Delle Ricerche, Parma, Italy; 3 Department of Computer Science, University of Milan, Milan, Italy; 4 Cognitive Architecture for Collaborative Technologies Unit, Italian Institute of Technology, Genova, Italy

**Keywords:** Neuroscience, Cognitive neuroscience, Social neuroscience, Human behaviour

## Abstract

People communicate using speech, gestures, and, less frequently, touches. An example of tactile communication is represented by handshake. Customs surrounding handshake vary in different cultures. In Western societies is mostly used when meeting, parting, as a sign of congratulations or at the end of a successful business. Despite its importance in social life, the neural mechanism underlying the affective components conveyed by handshake (“tactile vitality forms”) is unknown. Here we combined functional magnetic resonance imaging (fMRI) and electromyography (EMG), to investigate the neural affective activations during handshakes. We demonstrated that handshake conveying gentle or aggressive tactile vitality forms produces a stronger activation of the dorso-central insula. The simultaneous presence of emotional facial expressions modulates the activation of this insular sector. Finally, we provide evidence that the cingulate cortex is involved in the processing of facial expressions conveying different vitality forms.

## Introduction

Social interaction is defined as an exchange between two or more individuals. At its basis is the capacity to evaluate the communicative, affective meaning of others’ actions and gestures. Daniel Stern called these communicative aspects of gestures and actions vitality affects^1^ and subsequently vitality forms^2,3^. According to Stern^2^, five properties characterize vitality forms: time, space, force, trajectory and direction. Thanks to this movement gestalt, vitality forms play a double role in interpersonal interactions: the expression of vitality forms allows the agent to communicate his attitude, while the perception of vitality forms allows the receiver to understand the attitude of others.

It is important to stress from the outset that vitality forms are a concept completely different from emotions. Vitality forms represent the way (“the form”) with which an action is performed, regardless of whether the action is devoid of emotional content, so called “cold actions” like reaching, grasping, taking, giving, or it is emotionally laden. An example of the latter action type could be anger. Indeed, anger might be overtly expressed or be internalized; it might be cold or aggressive. In all these cases, the emotion is the same but its vitality forms, i.e. the form with which is expressed, is radically different.

In a series of previous studies, we investigated the neural bases of visual and auditory vitality forms^4–13^. We found that the observation of vitality forms of gentle or rude actions determines the selective activation of the dorso-central insula^4,5,12,13^. In these studies we also demonstrated that the dorso-central insula is activated during imagining as well as during the execution of actions conveying gentle or rude vitality forms^5^.

Besides observation of actions and gestures of others, individuals also interact using touch. A typical example is handshaking. Handshaking is a common greeting behavior in Western cultures. The handshake conveys information about the affective state and the personality of an individual^14,15^. A “good” handshake may reveal friendliness, sociability and dominance, whereas a “poor” handshake may reveal shyness, introversion, and neuroticism^14^. A gentle or aggressive handshake may also allow the receiver to understand whether the agent is a kind or a rude person, thus stressing the powerful role of handshaking in social interactions^16^. Considering that the definition of vitality form by Stern concerned specifically the visual aspects of actions, we will use here the term *tactile vitality forms* to describe the affective and communicative aspects of the handshake.

It is surprising that, notwithstanding the importance of the handshake in social interactions, there is no study describing the neural basis underlying the tactile vitality forms. The main aim of the present study is to fill this gap and to describe the brain structures involved in encoding handshake tactile vitality forms. The second aim is to investigate whether and how interpersonal context, conveyed by facial expression, can modulate these brain structures and, more specifically, whether the observation of a happy or angry facial expression could modify the activity of brain areas involved in the processing of tactile vitality forms, rendering the perception of a handshake more positive or more negative.

To investigate these issues, two fMRI experiments were carried out. Experiment 1 (Exp. 1) consisted of three situations. In the first, the experimenter with his right hand shook the right hand of participant, while the participant observed video clips showing dynamically facial expression (angry, neutral and happy). The handshake was performed in an aggressive, neutral, or gentle way and was congruent with the expression conveyed by the facial expression (e.g. gentle handshake and happy face, aggressive handshake and angry face). In the second situation, participants were presented with the same facial expressions, but did not receive a handshake. Finally, in the third situation, participants received only a handshake (aggressive, gentle or neutral), without the presentation of facial stimuli. In Experiment 2, participants were tested in the same three situations as in Exp. 1, except that the handshake was always performed in a neutral way.

The results showed that handshake conveying tactile vitality forms produced, relative to control, a strong activation of the dorso-central insula and of the middle cingulate cortex. The interpersonal social context, conveyed by facial expressions, modulated the activation of these two areas.

## Materials and methods

### Preliminary behavioral study

**Figure 1:**
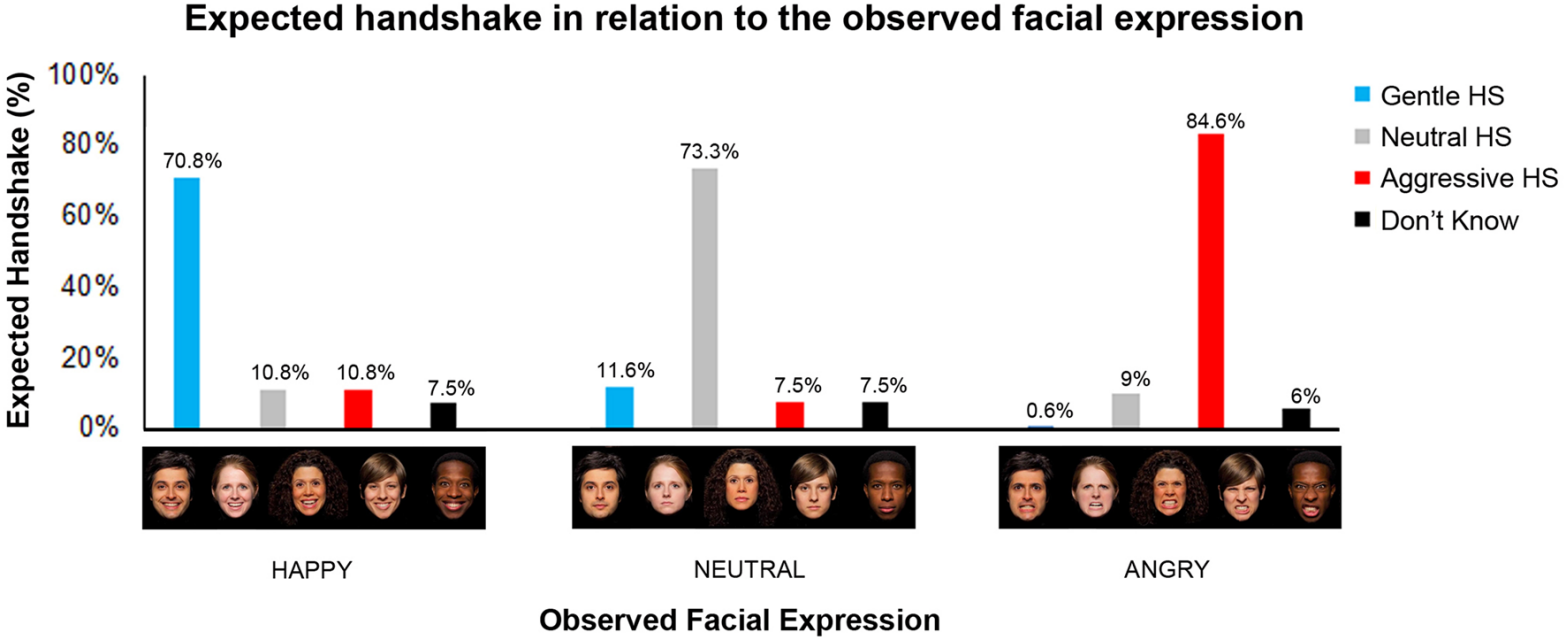
Graph shows the participants’ score indicating the type of handshake that they expected in association with the observation of happy, neutral and angry facial expressions. Under the graph is shown the observed facial expressions used to collect the participants’ responses. Facial expressions were freely shared by https://apprecs.com/ios/415011116/volafriends. Asterisks indicate statistical significance (p < 0.05).

A preliminary behavioral study, conducted before the two fMRI experiments, was carried out in order to ascertain what type of handshake participants expected in association with the observation of angry, happy and neutral facial expressions. Thirty healthy right-handed participants (20 females and 10 males, mean age = 24.8 years, SD = 2.5 years) took part in this behavioral study. Participants were presented with visual stimuli, consisting of three different facial expressions (angry, happy and neutral) performed by five actors (3 females and 2 males). In total 15 facial expressions were presented. Participants were required to observe the facial expression and indicate the type of handshake expected from that person on a bar showing four possible choices: “gentle”, “neutral”, “aggressive”, “don’t know”. Subsequently, we verified the presence of differences among category response frequencies using a one-way chi square test. This analysis indicated that participants observing happy, neutral and angry facial expressions, expected to receive a congruent handshake and namely a gentle (70.8%; one-sample chi-square test: χ^2^ (3) = 134,8, p = 0.001), neutral (73.3%; χ^2^ (3) = 150, p = 0.001) and aggressive (84.6%; χ^2^ (3) = 231,6, p = 0.001) handshake respectively (Fig. 1). Comparing males and females responses, no differences were found (see Supplementary Material, Fig. S1).

### fMRI study

#### Participants

Two fMRI experiments were carried out. The same participants (N = 15, six females and nine males, mean age = 24.6, SD = 2.6) took part in both experiments. The choice to use of the same participants in the two experiments was done to avoid that possible differences in neural activation patterns were due to individual participant differences. The Exp. 2 was carried out two months after the beginning of the Exp. 1. The choice to collect 15 subject in the current study is based on results provided by a power analysis carried out on previous fMRI data concerning the vitality forms^5,12^. Results of power analysis indicated that, in order to obtain a medium effect in the dorso-central insula due to the perception and expression of vitality forms, it is essential to collect a sample consisting of at least 15 participants [(partial eta square = 0.21, α = 0.05, β = 0.95 and non sphericity correction 0.7 (ε)]. All participants gave their written informed consent to participate in the Exp. 1 and Exp. 2. Both experiments were approved by the ethics committee of the University of Parma (UNIPRMR750v1) in accordance with the Declaration of Helsinki. All participants had normal or corrected-to-normal vision.

#### Paradigm and task: fMRI study

**Figure 2:**
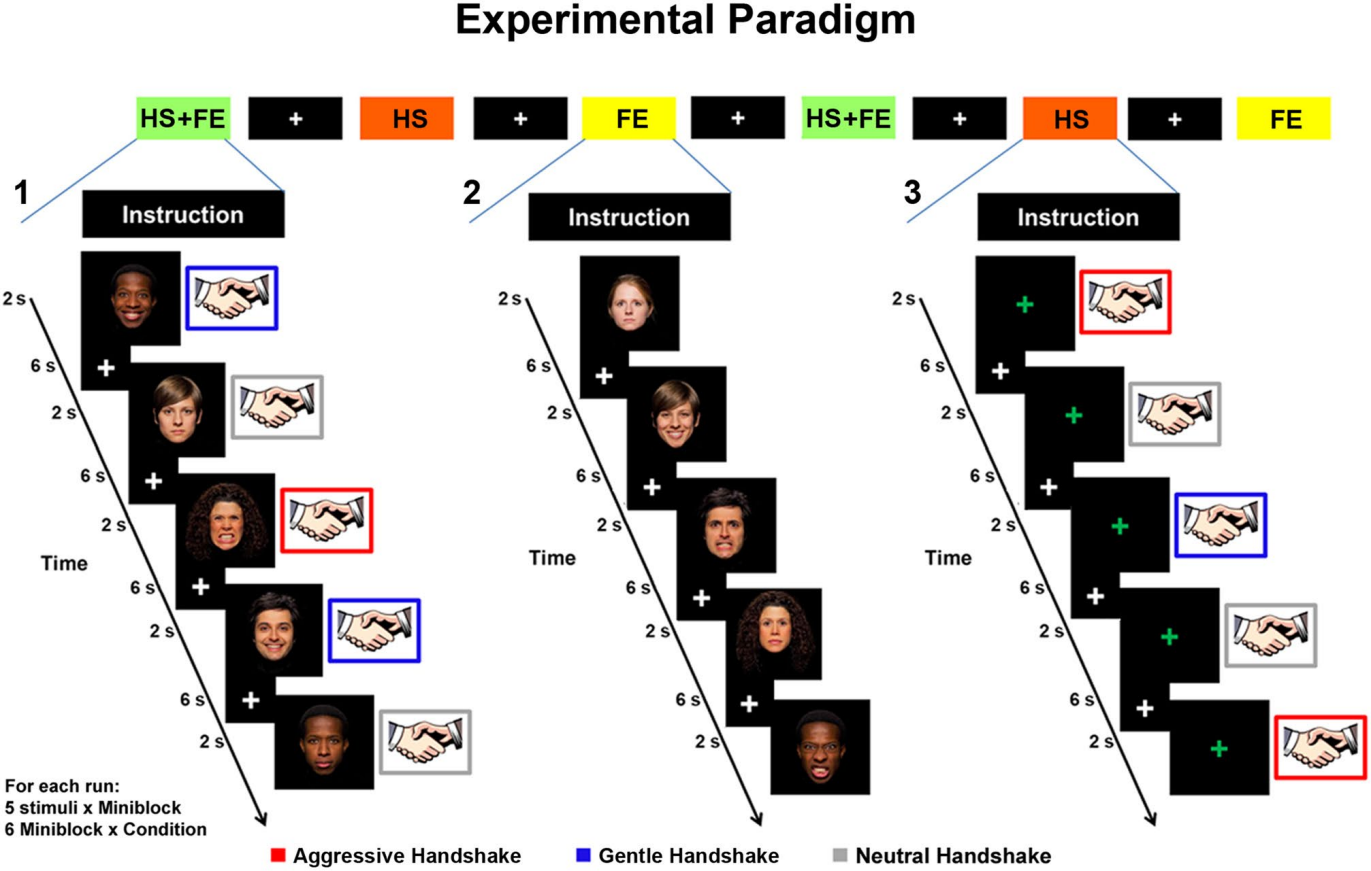
Experimental paradigm adopted in the experiment 1. Three different situations were presented [handshake and facial expression, HS + FE (1); facial expression, FE (2); handshake, HS (3)]. Each video stimulus was presented as a single event lasting 2 s in miniblocks composed of five stimuli intermixed with an inter stimulus interval lasting 6 s. During handshake and facial expression (HS + FE) and facial expressions (FE) situations, a total of 15 facial expressions were presented: 10 for the vitality condition (5 actors × 2 facial expressions) and 5 for the control condition (5 neutral expressions). Facial expressions were freely shared by https://apprecs.com/ios/415011116/volafriends. During the observation of happy, angry and neutral facial expressions, participants received a congruent handshake (gentle, blue color; aggressive, red color; neutral, gray color). During the *handshake* situation (3), participants received a gentle (blue color), aggressive (red color) and neutral (gray color) handshake. The same paradigm was also adopted in experiment 2 but during the observation of happy, angry, neutral facial expressions, participants received always a neutral handshake (for details see Figure S3).

Both fMRI experiments consisted of three different situations (handshake and facial expressions, HS + FE; facial expression, FE; handshake, HS). The facial expressions presented in the fMRI study were the same as those used in the preliminary study. In the first situation (HS + FE), participants were presented with video clips showing actors producing two dynamic facial expressions (happy and angry) and other video clips showing the same actors tilting the head to the left or right side while maintaining a neutral facial expression (control stimuli). It is important to note that, during the presentation of video clips, participants received a handshake given by the experimenter with his right hand (see Fig. 2). In the Exp. 1, the handshake strength was congruent with the presented facial expression and was performed in an aggressive or gentle manner (*tactile vitality forms* condition). Additionally, during the observation of the neutral facial expression, the handshake was performed in a neutral way (*control* condition). In contrast, in Exp. 2, during the first situation (HS + FE) participants received a handshake always performed in a neutral way (see Fig. S3). In both experiments, in the second situation (FE) participants were presented with video clips showing dynamic facial expressions (happy, angry, neutral) and did not receive a handshake (Fig. 2).

Finally, in both experiments, in the third situation (HS) participants received only a handshake without a facial expression. More specifically, in Exp. 1 participants received different types of handshakes (gentle, aggressive, neutral; Fig. 2), whereas in Exp. 2 they received only one type of handshake (neutral; see Fig. S3). It is important to note that, the dorso-central insula is endowed with motor properties and it is active during the execution of actions. Therefore, aiming to assess the insula activity when receiving *tactile vitality forms*, in order to exclude that the activation of this insular sector were merely due to the execution of handshake, we required participants to be completely passive and relaxed and not to return the handshake.

Each situation consisted of independent miniblocks. Each miniblock was composed by five stimuli and each stimulus lasted 2 s. Stimuli were intermixed by a rest period lasting 6 s (see Fig. 2). In Exp.1 and Exp. 2 five experimental conditions were presented (Exp. 1: Handshake (HS), Facial Expressions (FE), Aggressive HS and Angry FE, Gentle HS and Happy FE, Neutral HS and Neutral FE; Exp. 2: Handshake, Facial Expressions, Neutral HS and Angry FE, Neutral HS and Happy FE, Neutral HS and Neutral FE). For each condition six miniblocks were presented in a randomized order (30 single trials per condition). In both experiments, before each mini block, an instruction panel indicated to participants the type of situation that would be presented (Fig. 2). Randomly, in the 40% of cases, at the end of the experimental miniblock containing facial expressions (HS + FE, FE), participants had to indicate with the left hand the last presented facial expression (happy, angry, neutral) on a response box placed inside the scanner. The analysis of the catch trials showed that the participants’ mean response accuracy was 85% in Exp. 1 and 88.3% in Exp. 2.

#### fMRI data acquisition

Imaging data were collected on a 3 T Discovery MR750 GE scanner equipped with an eight-channel receiver head coil. Functional images were acquired using a gradient EPI sequence with a TR of 2000 ms, TE of 30 ms, flip angle of 90°, parallel imaging acceleration factor of 2, 205 × 205 mm^2^ field of view, and voxel size of 2.5 × 2.5 × 3 mm^3^. The scanning comprised three sequences composed by 266 ascending sequential volumes each. Then, each volume was composed by 40 axially slices. Additionally, a high resolution T1-weighted structural image (1 × 1 × 1 mm^3^) was acquired with a TR of 8100 ms, TE of 3.2 ms, and flip angle of 12° for each participant.

#### Statistical analysis

The data analysis for Exp. 1 and Exp. 2 was performed using SPM12 (Wellcome Trust Center for Neuroimaging, London, UK). The first three volumes of each run were discarded to allow T1 equilibration effects. For each participant functional data were anonymized. Then, functional volumes were first slice-timing corrected, realigned to the mean volume and unwarped for between-scan motion correction. Subsequently, the T1-weighted image was resampled into functional image space before segmentation into gray, white and cerebrospinal fluid and normalization to the Montreal Neurological Institute (MNI) space, according to the SPM12 preprocessing pipeline. Finally, spatial transformations derived from the segmentation step were then applied to the realigned EPIs for normalization to MNI space with a voxel size of 2 mm × 2 mm × 2 mm. At the end of preprocessing, all functional normalized volumes were then spatially smoothed with a 6-mm full-width half maximum isotropic Gaussian kernel. For all subjects, head motion was carefully checked and no participant met the exclusion criteria of 3 mm mean displacement. For both experiments, data were analyzed using a random-effects model^17^, implemented in a two-level procedure. At the first level (single-subject analysis), the BOLD signal was modeled using a general linear model (GLM) comprising the onset of each event, duration, and six motion parameters related to head motion for each functional run.

In Exp. 1, the GLM model consisted of seven regressors: *faces*, handshakes, gentle handshake and happy face, aggressive handshake and angry face, neutral handshake and neutral face, instruction and response. The stimuli were modeled as a single event lasting 2 s. The catch trial intermixed with experimental blocks was modeled as a single event lasting 6 s. In order to highlight the insular sector selective to vitality forms without the influence of the insular activation merely due to visual information, we subtracted at first level the general information of facial expressions (happy, neutral, angry). In this way, we obtained the following contrasts: aggressive handshake and angry face vs. faces (Aggressive Tactile Vitality Form), gentle handshake and happy face vs. faces (Gentle Tactile Vitality Form), neutral handshake and neutral face vs. faces (Neutral Tactile Vitality Form).

In the second-level analysis (group analysis), for each participant, the contrast images of the first level were entered into two flexible factorial models. The first model consisted of five regressors (faces, handshakes, gentle handshake and happy face, aggressive handshake and angry face, neutral handshake and neutral face) and considered the activation patterns vs. baseline. The second model consisted of three regressors (Aggressive Tactile Vitality Form, Gentle Tactile Vitality Form, Neutral Tactile Vitality Form) and considered the activation patterns resulting from the contrast among conditions (Aggressive Tactile Vitality Form vs. Neutral Tactile Vitality Form, Gentle Tactile Vitality Form vs. Neutral Tactile Vitality Form, Aggressive Tactile Vitality Form vs. Gentle Tactile Vitality Form).

In Exp. 2, the GLM model consisted of seven regressors: faces, handshakes, neutral handshake and happy face, neutral handshake and angry face, neutral handshake and neutral face, instruction and response. The stimuli were modeled as a single event lasting 2 s. The catch trial intermixed with experimental blocks was modeled as a single event lasting 6 s. As in Exp. 1, the general visual information of facial expressions was subtracted by the following contrasts: neutral handshake and angry face vs. faces (Tactile Vitality Form in Angry Context), neutral handshake and happy face vs. faces (Tactile Vitality Form in Happy Context), neutral handshake and neutral face vs. faces (Tactile Vitality Form in Neutral Context).

In the second-level analysis (group analysis), for each participant, the contrast images of the first level were entered into two flexible factorial models. The first model consisted of five regressors (faces, handshakes, neutral handshake and happy face, neutral handshake and angry face, neutral handshake and neutral face) and considered the activation patterns vs. baseline. The second model consisted of three regressors (Tactile Vitality Form in Happy Context, Tactile Vitality Form in Angry Context, Tactile Vitality Form in Neutral Context) and considered the activation patterns of the three conditions.

The location of the activation foci was determined in the stereotaxic space of the MNI coordinates system. All the activations revealed in the group analysis were identified using a statistical threshold of p < 0.001, FWE corrected at cluster level.

#### Testing for the vitality effect: region-of-interest analysis

On the basis of the functional maps obtained in Exp. 1, we carried out a conjunction analysis between the brain activations resulting from the contrasts Aggressive Tactile Vitality Form vs. Neutral Tactile Vitality Form and Gentle Tactile Vitality Form vs. Neutral Tactile Vitality Form to highlight the insular voxels selective for both aggressive and gentle *tactile vitality forms* (Fig. 4C). Then, in order to identify possible differences between aggressive and gentle *tactile vitality forms,* in the clusters resulting from the conjunction analysis in the left hemisphere (local maxima: X =  − 40 Y = 2 Z = 10) and in the right hemispheres (local maxima: X = 42, Y = 4, Z = 8), using the SPM Rex Toolbox (http://web.mit.edu/swg/rex), the BOLD signal change relative to three regressors (Aggressive Tactile Vitality Form, Gentle Tactile Vitality Form, Neutral Tactile Vitality Form) was extracted for each participant. In these ROIs, to avoid the problem of circularity in the analysis a comparison was made only between aggressive and gentle conditions.

In the Exp. 2, the functional maps obtained from the regressor *Tactile Vitality Form in Angry Context* highlighted the activation of the dorso-central insula and the cingulate cortex in the left hemisphere (local maxima: insula, X =  − 36, Y = 0, Z = 16; cingulum, X = − 6, Y = 0, Z = 44). In these two regions, using the SPM Rex Toolbox, the BOLD signal change relative to the neutral handshake received during the observation of different facial expressions (happy, neutral, angry) was extracted for each participant from the following regressors: Tactile Vitality Form in Happy Context, Tactile Vitality Form in Angry Context, and Tactile Vitality Form in Neutral Context. Then, for each region, the BOLD signal was modeled using a GLM comprising the participants’ BOLD activity relative to the neutral handshake received during the observation of happy, neutral and angry faces (contexts).

### Electromyography data acquisition and processing

EMG was used to monitor the handshake intensities during Exp. 1 and Exp. 2. Ag/AgCl EMG-electrodes (Falk Minow Services) were used in combination with the BrainAmp MR system (Brain Products GmbH, Munich, Germany) for the EMG signal recordings. Two electrodes were placed on the flexor muscle of the fingers of the right arm, 2 cm from each other, and arranged parallel to the direction of the muscle fibers. A reference electrode (ground) was placed on the elbow of the same arm. The skin under the electrodes was cleaned with alcohol solution and scrubbed to reduce electrical impedance. An electrolytic gel was placed between the electrode surface and the skin to facilitate the conduction of the electrical signal. All electrodes were attached to the BrainAmp amplifier (placed as far away from the scanner as possible), which converted the neurophysiological signals to digital signals. Signals were sampled by Brain Vision Recorder software with a sampling rate of 2500 Hz/channel and a resolution of 0.1 μV. The MRI scanner sent a trigger signal to the Brain Vision Recorder when a scan session started. Before the beginning of each trial, the experimenter received via digital audio system the instruction about the type of the handshake to perform (e.g. prepare to perform a gentle handshake). Then, after 2 s, again via digital audio system, the experimenter received information allowing him to start (go signal) and terminate (stop signal) the handshake in the established time (2 s). At the same time, participants were presented with video-clips showing the facial expression (2 s).

EMG signals were processed by using a homemade GUI of MATLAB R2018a (Mathworks, Natick, USA) and EEGLAB v14.1.2 toolbox (Swartz Center for Computational Neuroscience, CA, USA, https://sccn.ucsd.edu/eeglab/index.php)^18^.

The data were first corrected for scanner artifacts using the FMRIB tool of EEGLAB according to the method described by Allen^18^. Then, data were filtered using a 20–500 Hz pass-band filter and 50 Hz notch filter to minimize possible movements and power supply artifacts in the signal^19,20^. Thereafter, signals were rectified and epochs of 3000 ms corresponding to aggressive, gentle and neutral stimuli were extracted. A root mean square envelope over 200-sample intervals (80 ms) was performed to determine the mean amplitude of the EMG signal in each epoch. Finally, signals corresponding to the same stimuli were aligned and averaged. The intensities (mV/s) of aggressive, gentle and neutral handshakes were obtained by integrating the averaged signal in a time window of ± 750 ms around the peak of the signal. Finally, the mean EMG curves and intensities of each subject were averaged (for details of EMG data processing see Fig. S5).

## Results

### Experiment 1

#### Cortical activations vs. baseline in all three situations (HS, FE, HS + FE)

**Figure 3:**
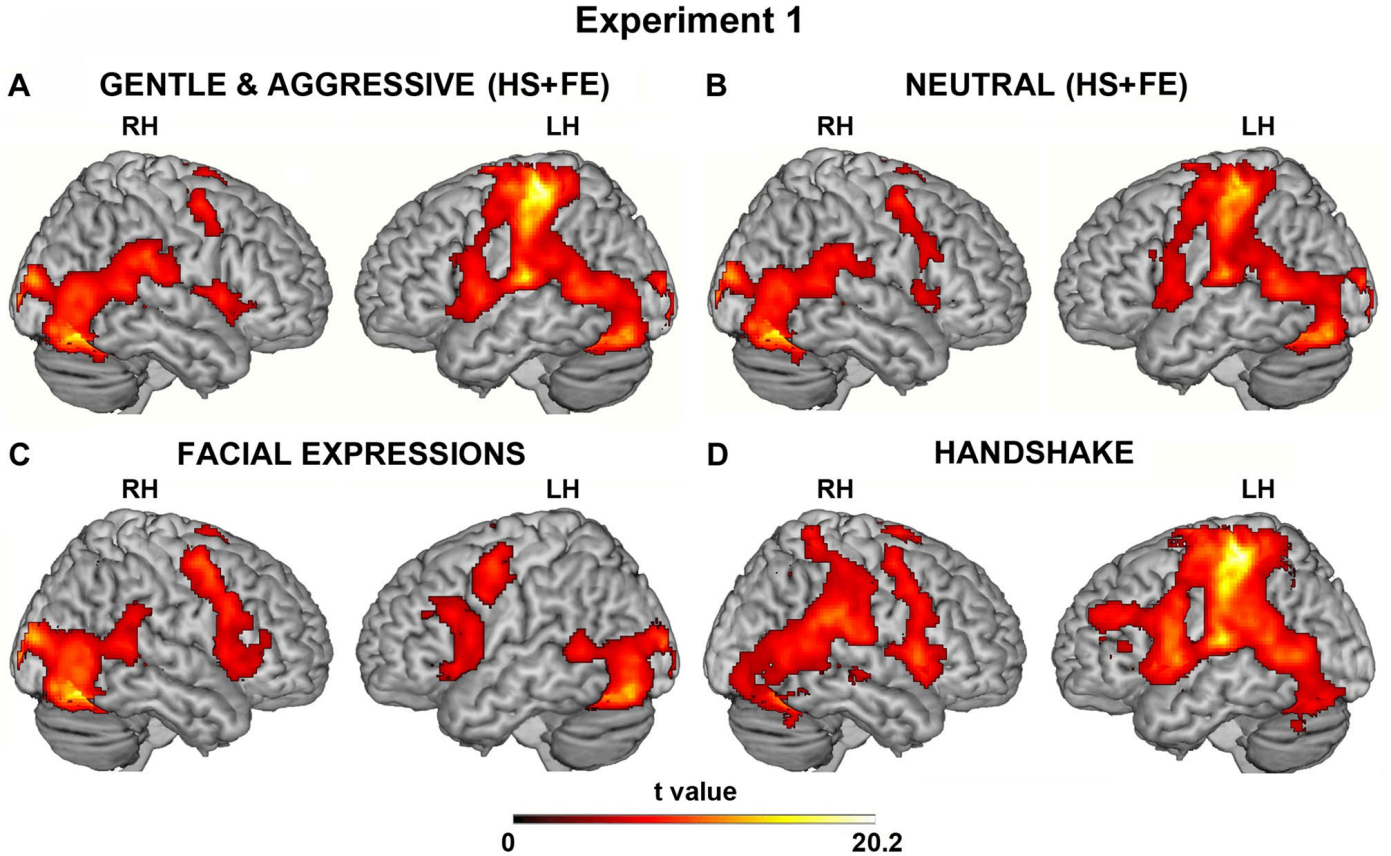
Brain activations resulting from the three different situations (handshake and facial expression, HS + FE; facial expression, FE; handshake, HS). Activations obtained in the first situation (HS + FE) during the *vitality forms* (aggressive and gentle) (**A**) and neutral (**B**) (control) conditions vs. baseline. Activations obtained in the second (FE) (**C**) and third situations (HS) (**D**) vs. baseline. These activations are rendered using a standard Montreal Neurological Institute brain template (P_FWE_ < 0.05 voxel level). LH, left hemisphere; RH, right hemisphere.

Figure 3A illustrates the brain activity resulting from aggressive and gentle handshakes vs. baseline during the observation of an angry or happy context. On the cortical surface, activations were present in the areas encoding somatosensory modality (SI and SII), in particular on the left hemisphere; the dorsal and ventral premotor areas; the posterior part of the temporo-parietal region and visual areas extending to the posterior part of the fusiform gyrus (for coordinates see Table 1). Additionally, activations were present, in the insula, the posterior part of the middle cingulate cortex, and the amygdala, bilaterally (see aldo Fig. S2). A similar, but weaker, cortical activation pattern was found during the control condition vs. baseline (neutral facial expression and neutral handshake; Fig. 3B, for coordinates see Table 1).

**Table 1:** Brain activations obtained in Exp. 1 from the three different situations (handshake and facial expression; facial expression; handshake).

Contrast of interest	Left hemisphere					Right hemisphere			
	x	y	z	Z-score		x	y	z	Z-score
**(A) Gentle and aggressive handshake vs. baseline (HS+FE)**									
Precentral gyrus	− 36	− 28	64	Inf	Fusiform gyrus	34	− 66	− 18	Inf
Postcentral gyrus	− 44	− 32	60	Inf	Rolandic operculum	50	4	8	7.24
Fusiform gyrus	− 38	− 54	− 20	Inf	Inferior frontal gyrus	52	12	2	7.23
Rolandic operculum	− 52	− 22	44	Inf	Amygdala	20	− 4	− 16	7.21
Insula (posterior short gyrus)	− 40	2	8	Inf	Middle frontal gyrus	48	0	54	7.20
Cerebellum	− 28	− 78	− 20	Inf	Insula	38	6	4	7.11
Hippocampus	− 22	− 28	− 6	Inf	Cerebellum	24	− 56	− 50	6.03
					Postcentral Gyrus	60	− 12	42	5.71
					Superior Parietal Lobule	24	− 46	72	5.41
					Putamen	24	6	6	5.01
					Pallidum	16	2	− 4	4.83
**(B) Neutral handshake vs. baseline (HS+FE)**									
Precentral gyrus	− 38	− 28	64	Inf	Fusiform gyrus	34	− 66	− 18	Inf
Postcentral gyrus	− 44	− 32	60	Inf	Middle frontal gyrus	50	0	54	7.33
Fusiform gyrus	− 36	− 56	− 20	Inf	Inferior frontal gyrus	50	14	2	6.07
Calcarine gyrus	− 2	− 82	− 6	Inf	Amygdala	20	− 4	− 16	7.28
Lyngual gyrus	− 2	− 78	− 4	Inf	Hippocampus	18	− 12	− 16	5.44
Cerebellum	− 28	− 78	− 20	Inf	Cerebellum	14	− 62	− 48	5.66
Inferior parietal lobule	− 30	− 50	40	5.39	Postcentral gyrus	62	− 12	40	5.63
					Putamen	24	6	6	5.50
					Superior parietal lobule	24	− 46	72	4.78
**(C) Facial expressions vs. baseline**									
Hippocampus	− 22	− 28	− 6	Inf	Fusiform gyrus	34	− 66	− 18	Inf
Precentral gyrus	− 50	− 2	52	7.58	Calcarine gyrus	14	− 96	2	Inf
Posterior medial frontal	− 2	8	48	7.05	Thalamus	20	− 30	2	Inf
Inferior frontal gyrus	− 48	18	24	6.79	Middle frontal gyrus	48	0	54	Inf
Inferior parietal lobule	− 30	− 54	48	5.98	Precentral gyrus	50	6	48	Inf
Thalamus	− 8	− 16	− 2	5.41	Middle cingulate cortex	8	14	38	6.85
Superior occipital gyrus	− 22	− 66	36	4.82					
Anterior cingulate cortex	− 6	8	28	4.76					
**(D) Handshake vs. baseline**									
Precentral gyrus	− 36	− 28	64	Inf	Cerebellum	34	− 58	− 22	Inf
Postcentral gyrus	− 44	− 32	60	Inf	Rolandic operculum	48	− 28	20	Inf
Rolandic operculum	− 40	− 22	− 18	Inf	Thalamus	14	− 28	2	7.34
Inferior parietal lobule	− 46	− 24	38	Inf	Pallidum	16	2	− 4	6.09
Insula (posterior short gyrus)	− 40	2	6	Inf	Middle cingulate cortex	12	− 24	36	5.59
Inferior frontal gyrus	− 52	8	12	Inf	Middle frontal gyrus	32	50	26	5.48
Cerebellum	− 10	− 74	− 46	5.51	Superior temporal gyrus	28	− 4	70	5.01
**(E) Aggressive tactile VF vs. neutral tactile VF**									
Precentral gyrus	− 36	− 28	64	Inf	Rolandic operculum	50	4	8	5.30
Middle cingulate cortex	− 2	0	42	6.09	Insula (posterior short gyrus)	44	6	4	5.24
Rolandic operculum	− 52	− 22	14	5.89	Inferior temporal gyrus	48	− 68	− 6	5.13
Insula (posterior short gyrus)	− 38	4	10	5.47					
Middel occipital gyrus	− 26	− 88	0	4.70					
**(F) Gentle tactile VF vs. neutral tactile VF**									
Precentral gyrus	− 36	− 28	64	Inf	Cerebellum	16	− 50	− 20	6.30
Postcentral gyrus	− 46	− 28	58	Inf	Insula (posterior short gyrus)	42	4	8	5.14
Middle cingulate cortex	− 4	− 8	48	5.75	Rolandic operculum	52	− 14	12	5.01
Insula (posterior short gyrus)	− 42	2	8	5.47					

Activations obtained in the first situation (HS + FE) during the *vitality forms* (aggressive and gentle) (A) and neutral (B) (control) conditions vs. baseline. Activations obtained in the second (facial expression) (C) and third situations (handshake) (D) vs. baseline. Activation resulting from aggressive vs. neutral (E) and gentle vs. neutral (F) contrasts. Local maxima, as shown in Figs. 2 and 3, are given in MNI standard brain coordinates, significant threshold was set at P_FWE_ < 0.05.

The observation of facial expressions (happy, angry, neutral) vs. baseline produced a strong activation of visual areas, particularly of the posterior part of the fusiform gyrus in the left and right hemispheres (Fig. 3C). There was also an activation of the dorsal and ventral premotor cortices, the anterior part of the insula, the posterior part of the middle cingulate cortex, and the amygdala bilaterally (for coordinates see Table 1).

Finally, the handshake per se (without context) performed in an aggressive, gentle and neutral manner, produced the activation of areas SI and SII on the left hemisphere; the dorsal and ventral premotor areas; the posterior part of the temporo-parietal region. There was a bilateral activation of the insula (*anterior* and *middle* sectors), the posterior part of the middle cingulate cortex extending to the anterior sector, and the amygdala (Fig. 3D; for coordinates see Table 1).

#### Contrast between aggressive and gentle tactile vitality forms vs. control (HS + FE)

**Figure 4:**
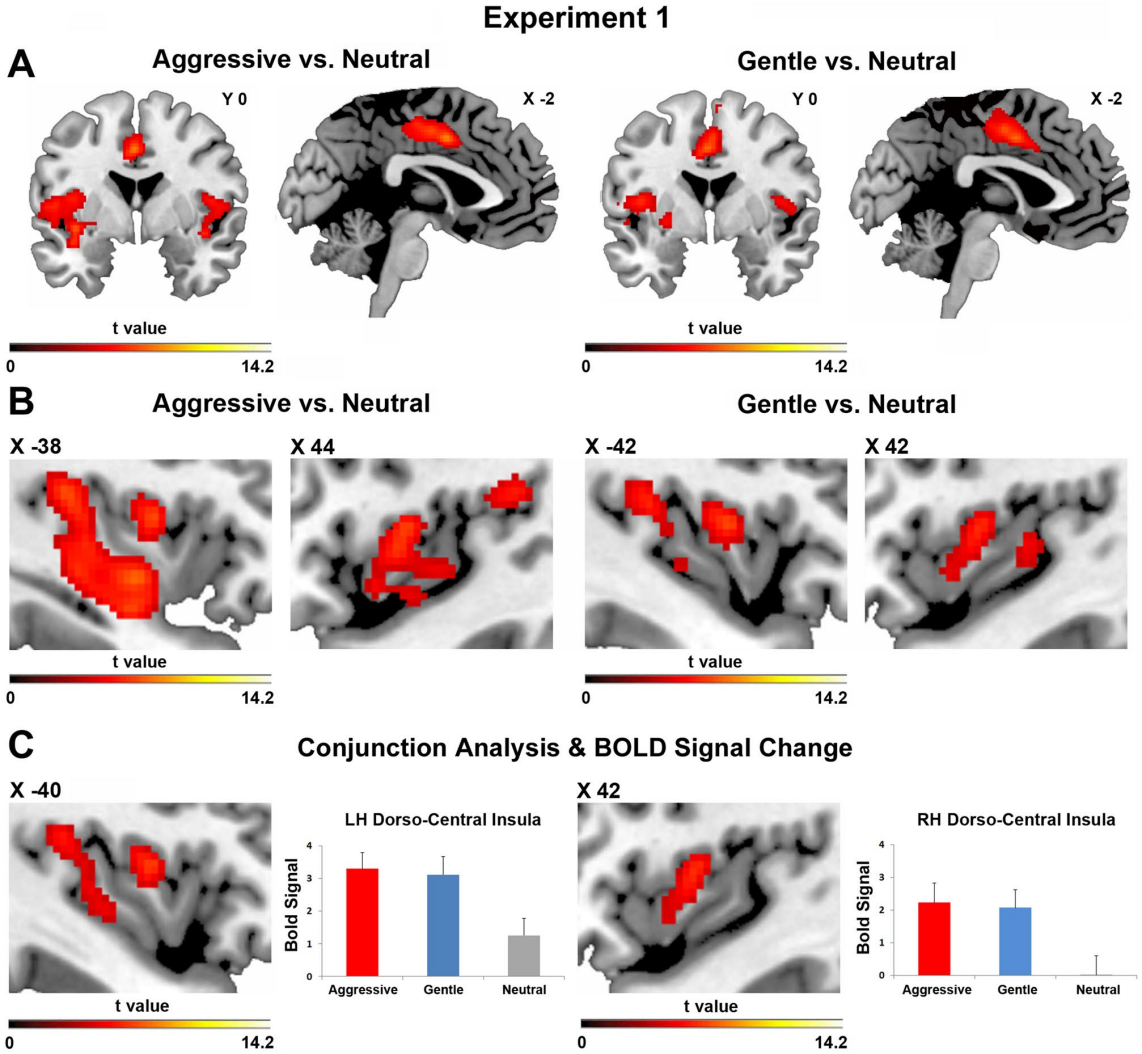
Parasagittal sections showing the activations of the insular and cingulate cortices obtained in situation 1 (handshake and facial expression) for the contrasts aggressive tactile vitality form vs. neutral tactile vitality form (aggressive vs. neutral) and gentle tactile vitality form vs. neutral tactile vitality form (gentle vs. neutral) (**A**,**B**). The conjunction analysis between the contrasts aggressive tactile vitality form vs. neutral and gentle tactile vitality form vs. neutral revealed a common activation of the dorso-central sector of insula (**C**). The bars presented on the right side indicate the BOLD signal change extracted from the left and right dorso-central insula. These brain activations are rendered using a standard Montreal Neurological Institute brain template (P_FWE_ < 0.05 at cluster level).

The contrast Aggressive Tactile Vitality Form vs. Neutral Tactile Vitality Form showed the activation of the middle cingulate cortex (Fig. 4A, left side), the dorso-central insula bilaterally (Fig. 4B), and areas SI and SII. The contrast Gentle Tactile Vitality Form vs. Neutral Tactile Vitality Form produced the activation of the same areas (Fig. 4A,B, right side; for coordinates see Table 1).

The conjunction analysis (Aggressive Tactile Vitality Form vs. Neutral and Gentle Tactile Vitality Form vs. Neutral) showed that the same insular sector was activated in both *aggressive* and *gentle* conditions relative to controls (Fig. 4C). On the basis of the functional maps obtained in the conjunction analysis, we extracted the BOLD signal change relative to the gentle, aggressive, and neutral conditions for each participant to identify possible differences in intensity between aggressive and gentle handshakes. The *t*-test revealed no significant difference between gentle and aggressive tactile vitality forms in the left and right hemispheres (Fig. 4C; *p* > 0.05).

#### EMG analysis

**Figure 5:**
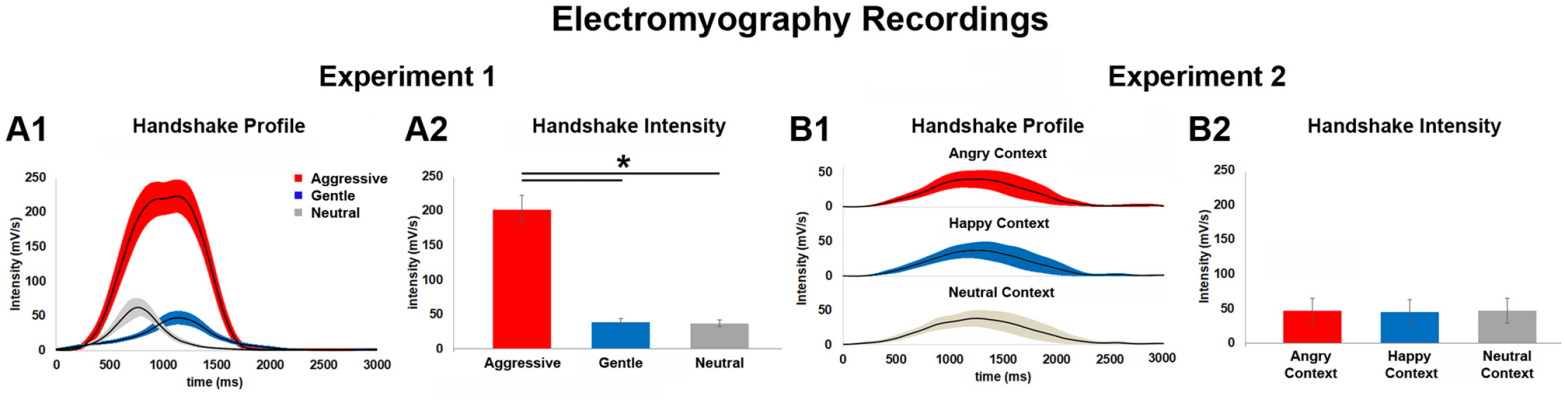
The graphs show the mean handshake profile (A1) and the mean intensity (**A2**) of the aggressive, gentle, and neutral handshakes detected by EMG in Exp. 1. The horizontal lines above the bars indicate the comparisons among aggressive, gentle, and neutral handshakes (**A2**). Asterisk indicates significant differences set at *p* ≤ 0.05 (*). The graphs show the mean handshake profile (**B1**) and the mean intensity (**B2**) of the neutral handshake detected by EMG in Exp. 2 during the observation of angry, happy and neutral facial expressions (context).

During the experiment, the strength of each handshake given by the experimenter to the participants was recorded using the EMG. The EMG analysis revealed that the intensity of the handshake changed as a function of the *tactile vitality form*. More specifically, during the execution of an aggressive handshake, the experimenter performed the action with a higher intensity than when performing the same action in a gentle or neutral way (Fig. 5A1). Differences between the intensities of aggressive, gentle and neutral handshakes are reported in Fig. 5A1,A2. The results showed significant difference (*p* ≤ 0.05) between aggressive and gentle handshakes and between aggressive and neutral handshakes (Fig. 5A2). No statistically difference in the EMG activity was observed between gentle and neutral handshakes (*p* = 0.7). Note that while the strength intensity was the same between gentle and neutral conditions, the shape of the two curves was different.

### Experiment 2

In Exp. 2, as in Exp. 1, participants were tested in three situations (HS + FE, FE, HS). In the first situation, they were presented with video clips showing dynamic facial expressions (happy, angry, neutral) while the experimenter simultaneously shook the participants’ right hand. In contrast to Exp. 1, the strength of the handshake was *always* performed in a neutral manner (Figure S3). The main aim of Exp. 2 was to evaluate the role of the facial expressions in the modulation of *tactile vitality forms* conveyed by the handshake. Concerning the cortical and subcortical activations obtained during the three situations vs. baseline, the results of Exp. 2 were very similar to those of Exp. 1. The main effects are shown in the Supplementary Material (Fig. S4; for coordinates see Table 2).

**Table 2:** Brain activations obtained in Exp. 2 from the three different situations (handshake and facial expression; facial expression; handshake).

Contrast of interest	Left hemisphere					Right hemisphere			
	x	y	z	Z − score		x	y	z	Z-score
**(A) Gentle and aggressive handshake vs. baseline (HS+FE)**									
Precentral gyrus	− 38	− 28	64	Inf	Fusiform gyrus	32	− 74	− 16	Inf
Postcentral gyrus	− 44	− 32	50	Inf	Cerebellum	14	− 66	− 48	7.39
Fusiform gyrus	− 28	− 78	− 18	Inf	Middle frontal gyrus	50	4	54	7.29
Supramarginal gyrus	− 52	− 22	16	Inf	Precentral gyrus	48	10	10	5.54
Lyngual gyrus	− 6	− 84	− 8	Inf	Amygdala	20	− 4	− 16	6.52
Cerebellum	− 22	− 74	− 18	Inf	Inferior frontal gyrus	54	10	6	5.27
Thalamus	− 14	− 26	2	7.82					
**(B) Neutral handshake vs. baseline (HS+FE)**									
Precentral gyrus	− 38	− 28	64	Inf	Fusiform gyrus	32	− 72	− 18	Inf
Lyngual gyrus	− 6	− 84	− 8	Inf	Middle frontal gyrus	50	4	54	7.50
Middle occipital gyrus	− 26	− 64	38	Inf	Amygdala	20	− 4	− 16	7.01
Inferior parietal lobule	− 28	− 50	40	5.38	Insula	46	6	32	6.26
Middle frontal gyrus	− 28	− 44	20	5.28	Thalamus	8	− 20	− 2	5.74
					Inferior frontal gyrus	52	10	6	5.24
**(C) Facial Expressions vs. baseline**									
Lyngual gyrus	− 6	− 84	− 8	Inf	Fusyform gyrus	32	− 74	− 16	Inf
Precentral gyrus	− 50	0	52	7.78	Calcarine gyrus	14	− 94	2	Inf
Posterior medial frontal	− 2	12	48	6.90	Middle frontal gyrus	50	4	54	Inf
Hippocampus	− 22	− 28	− 6	7.07	Posterior medial frontal	2	6	62	7.67
Thalamus	− 6	− 22	− 2	5.09	Insula	34	28	6	6.83
Insula	− 32	18	6	5.88	Inferior frontal gyrus	40	14	26	6.44
Temporal pole	− 52	8	− 2	5.70	Thalamus	8	− 22	− 2	5.42
Amygdala	− 20	− 6	− 16	5.62					
Inferior parietal lobule	− 32	− 56	52	5.34					
**(D) Handshake vs. baseline**									
Precentral gyrus	− 38	− 28	64	Inf	Lyngual gyrus	22	− 52	− 22	Inf
Postcentral gyrus	− 44	− 32	50	Inf	Fusiform gyrus	34	− 58	− 20	Inf
Supramarginal gyrus	− 52	− 22	− 16	Inf	Rolandic operculum	48	− 28	− 20	Inf
Thalamus	− 14	− 24	4	Inf	Insula	38	− 12	− 6	5.83
Amygdala	− 22	− 10	− 10	5.91	Middle frontal gyrus	50	4	54	5.74
					Inferior frontal gyrus	52	10	6	5.64
					Thalamus	8	− 16	− 2	5.62
**(E) Aggressive tactile VF in angry context**									
Postcentral gyrus	− 40	− 28	62	Inf	Cerebellum	22	− 50	− 22	7.52
Precentral gyrus	− 42	− 16	62	7.82	Rolandic operculum	48	− 26	20	5.38
Thalamus	− 14	− 26	4	5.48					
Middle cingulate cortex	− 8	− 28	46	5.20					
Insula (posterior short gyrus)	− 36	0	16	4.78					
**(F) Gentle tactile VF in happy context**									
Postcentral gyrus	− 40	− 28	62	Inf	Cerebellum	20	− 50	− 22	7.34
Precentral gyrus	− 30	− 20	72	7.11	Rolandic operculum	48	− 28	20	4.86
Rolandic operculum	− 50	− 22	16	7.07					
Insula (posterior long gyrus)	− 34	− 18	10	6.45					
Inferior parietal lobule	− 56	− 22	40	6.18					
**(G) Neutral tactile VF in neutral context**									
Postcentral gyrus	− 40	− 28	62	Inf	Cerebellum	22	− 50	− 22	7.63
Precentral gyrus	− 44	− 30	52	Inf	Rolandic operculum	46	− 28	20	5.38
Rolandic operculum	− 50	− 22	16	7.43					
Insula (posterior long gyrus)	− 34	− 18	10	6.84					
Inferior parietal lobule	− 44	− 26	40	6.34					

Activations obtained in the first situation (HS + FE) during the *vitality forms* (aggressive and gentle) (A) and neutral (B) (control) conditions vs. baseline. Activations obtained in the second (facial expression) (C) and third situations (handshake) (D) vs. baseline. Activation resulting from the following direct contrasts: aggressive vs. facial expressions (E), gentle vs. facial expressions (F), neutral vs. facial expressions (G). Local maxima, as shown in Fig. 4 and Fig. S4, are given in MNI standard brain coordinates, significant threshold was set at P_FWE_ < 0.05.

#### Comparison among angry, happy and neutral facial expressions during neutral handshaking

**Figure 6:**
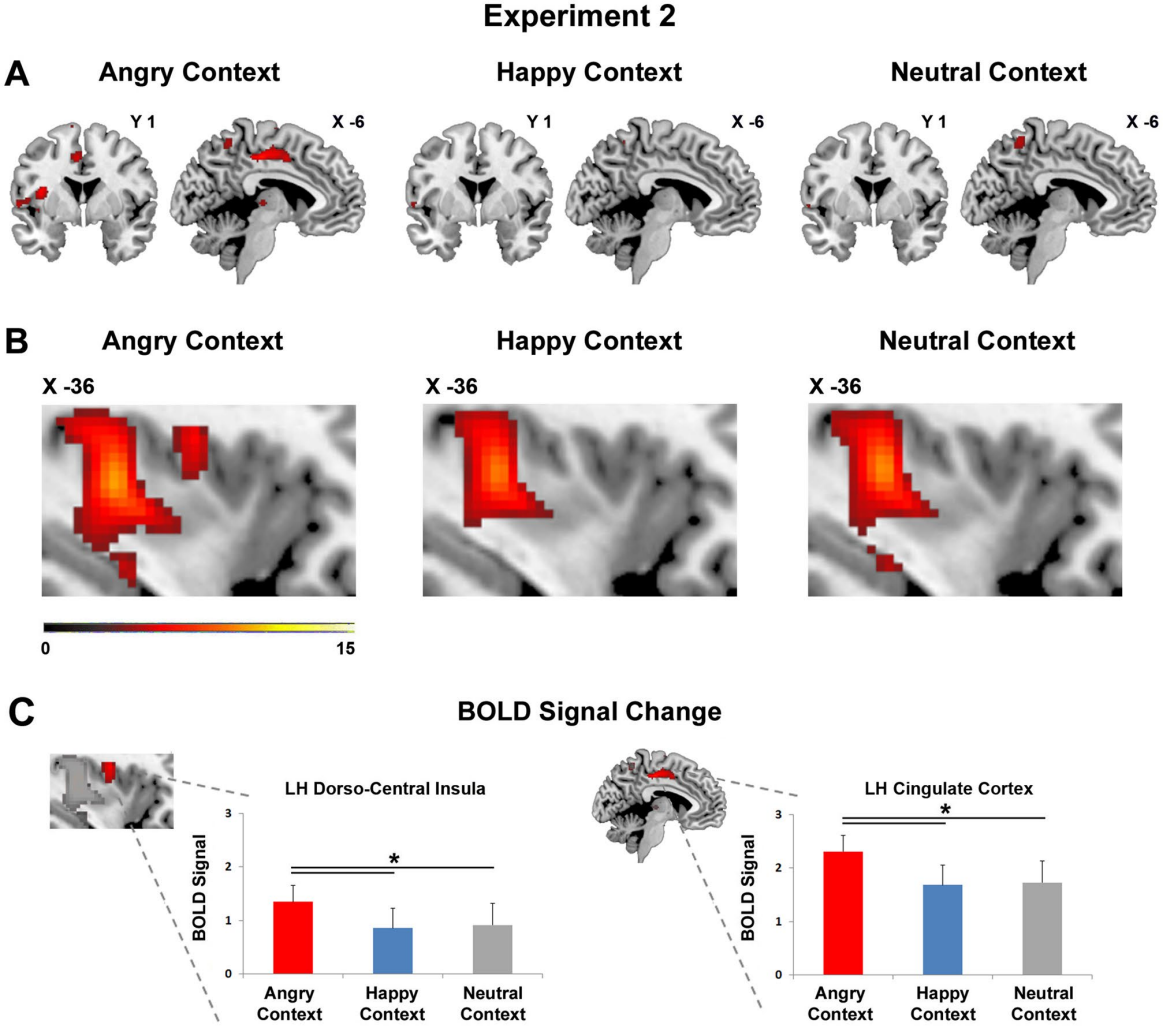
Parasagittal sections showing the activations of the insular and cingulate cortices modulated by the dynamic facial expressions. These brain activations are relative to the perception of handshakes obtained in situation 1 (handshake and facial expression) during the angry (Tactile Vitality Form in Angry Context), happy (Tactile Vitality Form in Happy Context), and neutral (Tactile Vitality Form in Neutral Context) contexts (**A,B**). The bar graph indicates the BOLD signal change extracted from the cluster of the left dorso-central insula and the cingulate cortex highlighted from the contrast *Tactile Vitality Form in Angry Context* vs. baseline (**C**). The horizontal line above the bars indicates the comparisons of the insula and cingulum activity during the handshake perception during the *angry, happy,* and *neutral* contexts. Asterisks indicate significant differences set at *p* ≤ 0.05 (*). The vertical lines indicate the standard error of the means (SEM). These brain activations are rendered using a standard Montreal Neurological Institute brain template (P_FWE_ < 0.05).

**Figure 7:**
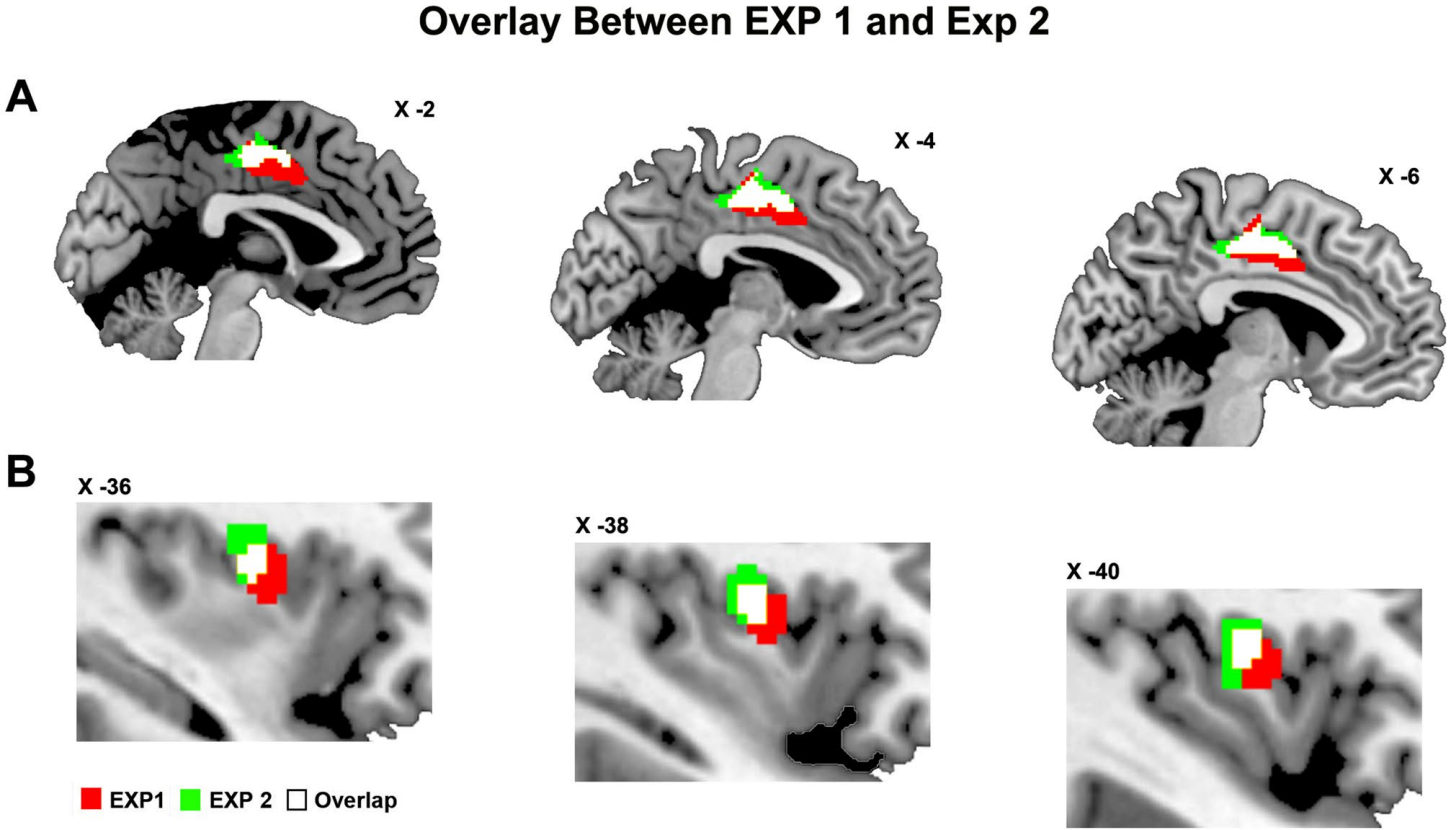
Parasagittal sections showing the activations of the insular and cingulate cortices selective for tactile vitality forms. The picture shows the brain activations involved in the processing of tactile vitality forms obtained in situation 1 (handshake and facial expression), and namely, the sector of the left cingulate cortex (**A**) and the left dorso-central insula (**B**). Red color indicates areas resulting from the conjunction analysis carried out in Exp. 1 between the contrasts *aggressive vs. neutral* and *gentle vs. neutral* while green color indicates areas found activated in Exp. 2 during the angry context. White color indicates the overlap area. These brain activations are rendered using a standard Montreal Neurological Institute brain template (P_FWE_ < 0.05).

In order to assess the effect of the social context (facial expressions) on the *tactile vitality forms* conveyed by a neutral handshake, we analysed and compared the brain activations of the handshake obtained during the observation of happy, angry and neutral facial expressions (Tactile Vitality Form in Happy Context vs. baseline, Tactile Vitality Form in Angry Context vs. baseline, Tactile Vitality Form in Neutral Context vs. baseline). The results showed a modulation of the brain activation due to the facial expression. In particular, the handshake, received during the observation of an angry facial expression, determined the activity of the posterior part of the middle cingulate cortex (Fig. 6A) and of the left dorso-central insula (Fig. 6B; for coordinates see Table 2). In contrast, these activations were absent when the handshake was received during the observation of a happy and neutral facial expression (Fig. 6A,B). Furthermore, in all three facial contexts, there was an activation of the posterior part of the insula and namely of the long gyri. Additionally, the analysis of the BOLD signal of the dorso-central insula and the cingulate cortex (versus baseline) showed different modulations during angry, happy, or neutral facial context (Fig. 6C). Notably, these insular and cingulate sectors partially overlaps with those found in Exp. 1 involved in the processing of *tactile vitality forms* (Fig. 7).

#### Region-of-interest analysis

The results of the GLM analysis carried out in Exp. 2 on the BOLD signal change extracted in the insular and cingulate cortices, indicated in both areas a significant difference of the BOLD signal among the happy, neutral and angry interpersonal contexts (insula: F = 4.2, *p* = 0.02; cingulum: F = 5.8, *p* = 0.01). Post hoc analysis revealed in both regions a significant difference between the angry and happy contexts and between the angry and neutral contexts (insula: *angry* > *neutral, p* = 0.03; *angry* > *happy, p* = 0.03; cingulum: *angry* > *neutral, p* = 0.02; *angry* > *happy, p* = 0.01; Newman–Keuls correction; Fig. 6C).

#### EMG analysis

As in Exp. 1, in Exp. 2 the strength of each handshake performed by the experimenter was recorded using EMG. The EMG analysis revealed no statistical differences (*p*-value ≤ 0.05) between the intensities of the neutral handshake performed in the happy, neutral and angry context (Fig. 5B).

## Discussion

In all actions, one can recognize two complementary aspects: the goal of the action (e.g., grasping an object, holding it, giving it to another person) and the form with which the action is executed (e.g., gentle, rude, violent). The importance of the forms of actions in social behavior has been extensively studied by Stern, who called them “vitality forms”^2^.

In pathological conditions, the two aspects of actions can become dissociated. For example, children with autistic spectrum disorder (ASD) often fail to understand the vitality forms with which the action is performed^21,22^, while they do not have difficulties in recognizing the goal of an observed action^20^. Recent analysis of action kinematics of ASD children showed that, although ASD children perform goal-directed actions pretty well, the way with which they perform them (vitality forms) are different from those of typically developing children^23^. As consequence, normotypical adults have problems in recognizing the vitality forms of ASD actions ^24^.

It is plausible to hypothesize that the behavioral deficit observed in children with ASD during vitality forms processing could be ascribed to an incorrect functioning of brain structures such as the insula in particular. In line with this hypothesis, various studies reported structural and functional alterations in the insula in individuals with ASD. In particular, alterations of gray matter volume in the insula have been reported in individuals with ASD^25–27^. Furthermore, a meta-analysis by Di Martino et al.^28^, reported a hypo-activation of the anterior insula in individuals with ASD relative to individuals with TD during the execution of different social tasks. However, one cannot exclude the involvement or cortical/subcortical areas functionally connected with it^29^.

Which are the neural structures underlying vitality forms? In a series of fMRI studies Di Cesare and colleagues investigated the cortical activations during the observation of transitive (goal-directed) and intransitive actions (gestures) expressed with evident vitality forms (e.g. gentle or rude)^4–7^, and during listening to action verbs also expressed with clear vitality forms^8,10,11^. In these studies, the authors found, as expected, an activation of the cortical areas encoding vitality forms conveyed by two sensory modalities. Most importantly, there was a selective activation of the dorsal-central insula in the contrast of actions/words conveying vitality forms vs. controls devoid of evident vitality forms. Subsequently, the same group showed that this insular sector is endowed with mirror properties and became active not only during the perception but also during the expression of vitality forms^5,11^.

In the present study, we assessed the neural basis of vitality forms conveyed by handshaking. The results showed that the handshakes performed in an aggressive or gentle way produced, relative to a neutral control, a stronger activation of the dorso-central insula in both hemispheres (see Fig. 4).

We studied handshaking not only in order to establish whether also tactile vitality forms (e.g., gentle or aggressive), modulated the activity of the dorso-central insula, but also to assess whether interpersonal context modified the insular activity during the handshake. Handshaking is a fundamental social gesture conveying the attitude of one individual towards another. When people offer their hand, they also search for certain social cues conveyed by the recipient such as the facial expression in order to better understand the attitude of others towards them. To clarify this point, we carried out a further fMRI experiment, in which a group of participants received a neutral handshake and were presented with video clips showing different facial expressions (happy, neutral and angry).

The results showed that the angry facial expression, significantly modulated the insula activity, which overlapped whit the one found in Exp. 1 and selectively involved in the processing of tactile vitality forms (Fig. 7B). These findings indicate that the dorso-central insula is the center encoding tactile vitality forms conveyed by a handshake (Exp. 1) and that its activity is modulated by the interpersonal context (Exp. 2). Our data are in line with the results of Ravaja et al.^31^ who showed that the observation of different facial expressions (angry, happy, sad and fear) performed by an avatar modulates the perception of the tactile stimulus intensity. Similarly, Ellingsen et al.^31^, found that the pleasantness of touch increased or decreased according to the concomitant observation of happy or angry facial expressions.

It is interesting to note that, differently from our previous fMRI experiments on vitality forms, in both Exp. 1 and Exp. 2 we also observed a strong activation of the posterior insula (long gyri). This is in agreement with experiments on pleasurable touch in humans, which showed that the stimulation of hand skin with a brush moving with a specific velocity activated the posterior insula^32,33^. This insular sector is in fact the target of afferent tactile C fibers (CT fibers)^34–37^. The CT fibers are a type of unmyelinated afferent nerve that carry signals from the receptive fields in the epidermis of mammalian hairy skin^38–42^. These fibers are sensitive to a caress-like range of stroking speeds associated with hedonically positive subjective responses^41,43^. Note that in the present study, the actor started the handshake gesture slowly, almost caressing the recipient’s right hand. This was true for the gentle, aggressive, and neutral conditions. It is likely that this “caressing” procedure was responsible for the activation of the posterior insula.

While the posterior insula is the target of CT fibers carrying out signals of affective touch, the primary somatosensory cortex (SI) is the target of myelinated fibers encoding tactile discriminative information. These findings indicate that affective and discriminative touch represent two distinct features of the tactile modality, processed in different cortical areas via two different pathways: lemniscal and extralemniscal. This issue was addressed by Morrison in a meta-analysis based on 17 fMRI studies carried out on the affective touch^44^. This meta-analysis revealed three important results: discriminative touch activates the primary somatosensory cortex (SI); both affective and discriminative touch produce the activation of the secondary somatosensory cortex (SII); the posterior part of the insula including the dorsal-central insula is a fundamental core involved in the processing of the affective touch^44^.

Another finding of the present study was the activation of the posterior part of the middle cingulate cortex during the administration of aggressive and gentle handshakes relative to control (Exp 1). Gothard and colleagues suggested that, in the monkey, the execution of facial expression is controlled via two motor sectors of the middle cingulate cortex and in particular via motor area M4^45^. Our data, in humans, are in line with these findings showing that a region of the middle cingulate cortex, corresponding to the border between the anterior and posterior middle cingulate cortex, is active during the observation of facial expression conveying emotions and in particular the negative ones (angry face) (see Fig. 6). According to Gothard and colleagues, area M4 is involved in the emotional control of facial expression. Note that, as mentioned in “Introduction” section, also the emotions can be expressed with different vitality forms. Indeed, anger might be expressed by the face with different vitality forms showing a cold wrath or an aggressive anger. In all these cases the emotion is the same, but the form with which it is expressed is different. We propose that the cingulate sector corresponding to area M4 is involved not so much in emotion but in expressing facial vitality forms. In agreement with Gothard, emotions are mostly encoded in the amygdala, which receives intereroceptive and other subcortical information concerning positive and negative social context. Then, it projects to the cingulate cortex that transforms this affective input into an appropriate facial expression selecting the set of muscle necessary for this expression.

An interesting question is to understand how the emotional facial expression may modulate the tactile vitality forms conveyed by handshake. Anatomical studies carried out in monkeys^46–49^, showing that the middle cingulate cortex is anatomically richly connected with the insula suggesting that, in humans, these two areas may be involved in a functional interaction during the expression and recognition of vitality forms. In humans, the anatomical connections between the cingulate and insula cortices are being investigated by our group.

In conclusion, our study provides three main findings. First, we demonstrated that a handshake conveying gentle or aggressive tactile vitality forms produces a stronger activation relative to control (neutral) of the dorso-central insula. Second, the simultaneous presence of emotional facial expressions modulates the activation of this insular sector during the handshake. Finally, we provide first evidence that the cingulate cortex is involved in the processing of vitality forms conveyed by facial expression.

## Supplementary Information

Supplementary Information.
