# Supplementary material for: The neural bases of tactile vitality forms and their modulation by social context
Source: Sci Rep. 2021 Apr 27;11:9095. doi: 10.1038/s41598-021-87919-z (PMC8079712; doi:10.1038/s41598-021-87919-z)
Supplement: Supplementary file 1 — Supplementary Information. [file 41598_2021_87919_MOESM1_ESM.pdf]

Supplementary Materials for

The neural bases of tactile vitality forms and their modulation by social context

G. Rizzolatti[1,2]*, A. D'Alessio[2], M. Marchi[3], G. Di Cesare[1,4]

1 Department of Medicine and Surgery, Neuroscience Unit, University of Parma, Parma, Italy;

2 Istituto di Neuroscienze, Consiglio Nazionale delle Ricerche, Parma, Italy.

3 Department of Computer Science, University of Milan, Milan, Italy;

4 Italian Institute of Technology, Cognitive Architecture for Collaborative Technologies Unit, Genova (Italy);

***Corresponding author**

giacomo.rizzolatti@unipr.it

Considerations on gender differences in behavioural and fMRI experiments.

In order to exclude the possibility that the ratio of female to male may affect behavioral and fMRI results, we tested possible differences between males and females by carrying out two additional analyses: one concerned the preliminary study and one relative to the fMRI study. Specifically, in the preliminary behavioral study, we compare the males and females correct responses concerning the type of handshake that they expected in association with the observation of happy, neutral and angry facial expressions. The result of this analysis shows that, between males and females responses, there is not a significant difference (Mann Whitney test).

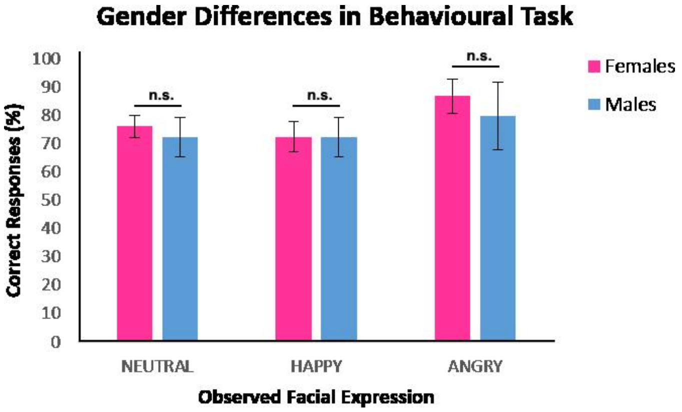

Figure S1. Comparison between males and females correct responses indicating the type of handshake that they expected in association with the observation of happy, neutral and angry facial expressions. The Mann Whitney test indicate that there is not significant effect between males and females in all three conditions (Neutral, Happy, Angry).

Additionally, to avoid the possibility that the insula and cingulum activity observed in our study may be affect to the gender, we carried out a new fMRI analysis on six males and six females separately. The results showed that in both males and females, the processing of tactile vitality forms produced a significant activation of the insular and cingulate cortices in the left hemisphere.

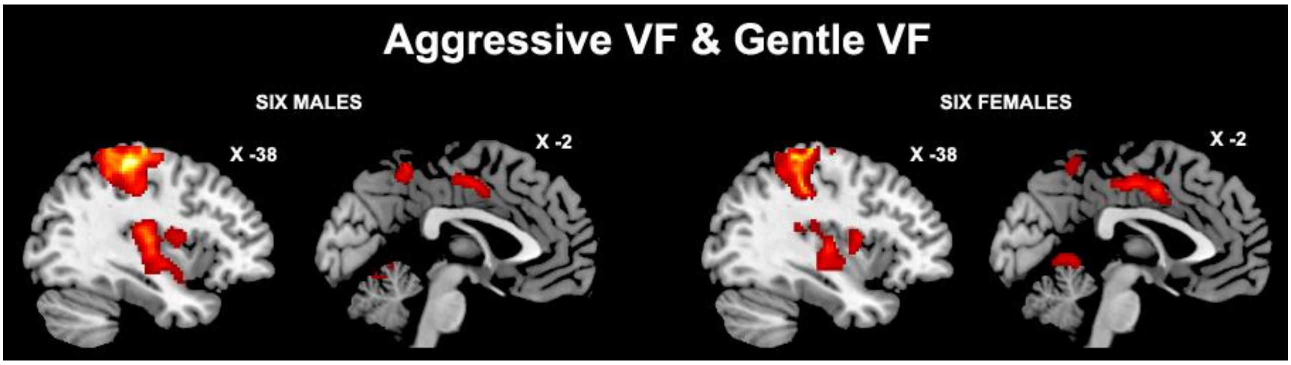

Figure S2. Comparison between brain activations obtained with six males and six females (threshold set at p<0.05 FWE at cluster level).

Additional Figures.

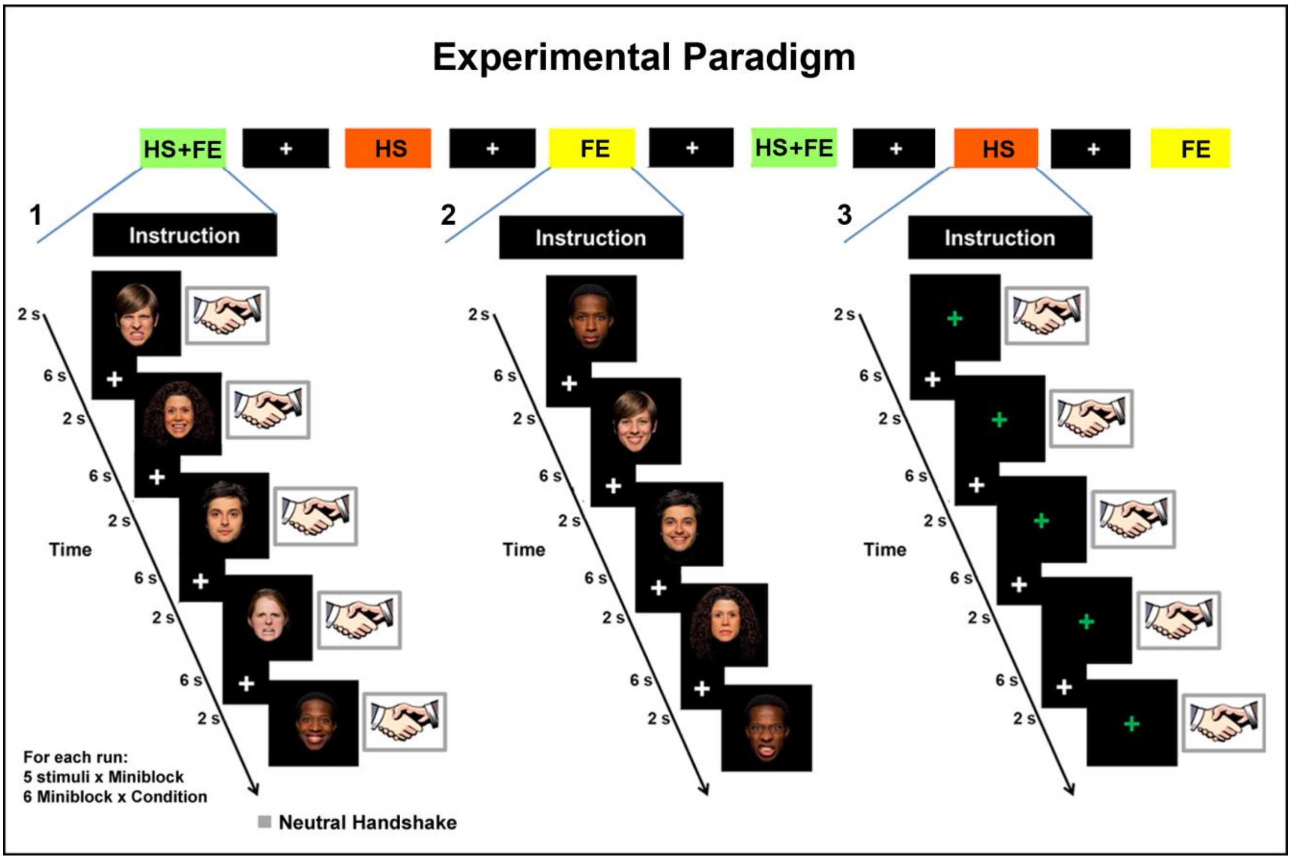

Figure S3. Experimental paradigm adopted in the experiment 2. Three different situations were presented [*handshake & facial expression*, HS+FE (1)*; facial expression*, FE (2)*; handshake*, HS (3)]. Each video stimulus was presented as a single event lasting 2s in miniblocks composed of five

stimuli intermixed with an inter stimulus interval lasting 6 s. During *handshake & facial expression* (HS+FE) and *facial expression* (FE) situations, a total of 15 facial expressions were presented: 10 for the vitality condition (5 actors x 2 facial expressions) and 5 for the control condition (5 neutral expressions). During the observation of happy, angry, neutral facial expressions, participants received always a neutral handshake (gray color). During the *handshake* situation (3), participants received a neutral (gray color) handshake without any visual context. Facial expressions were freely shared by https://apprecs.com/ios/415011116/volafriends

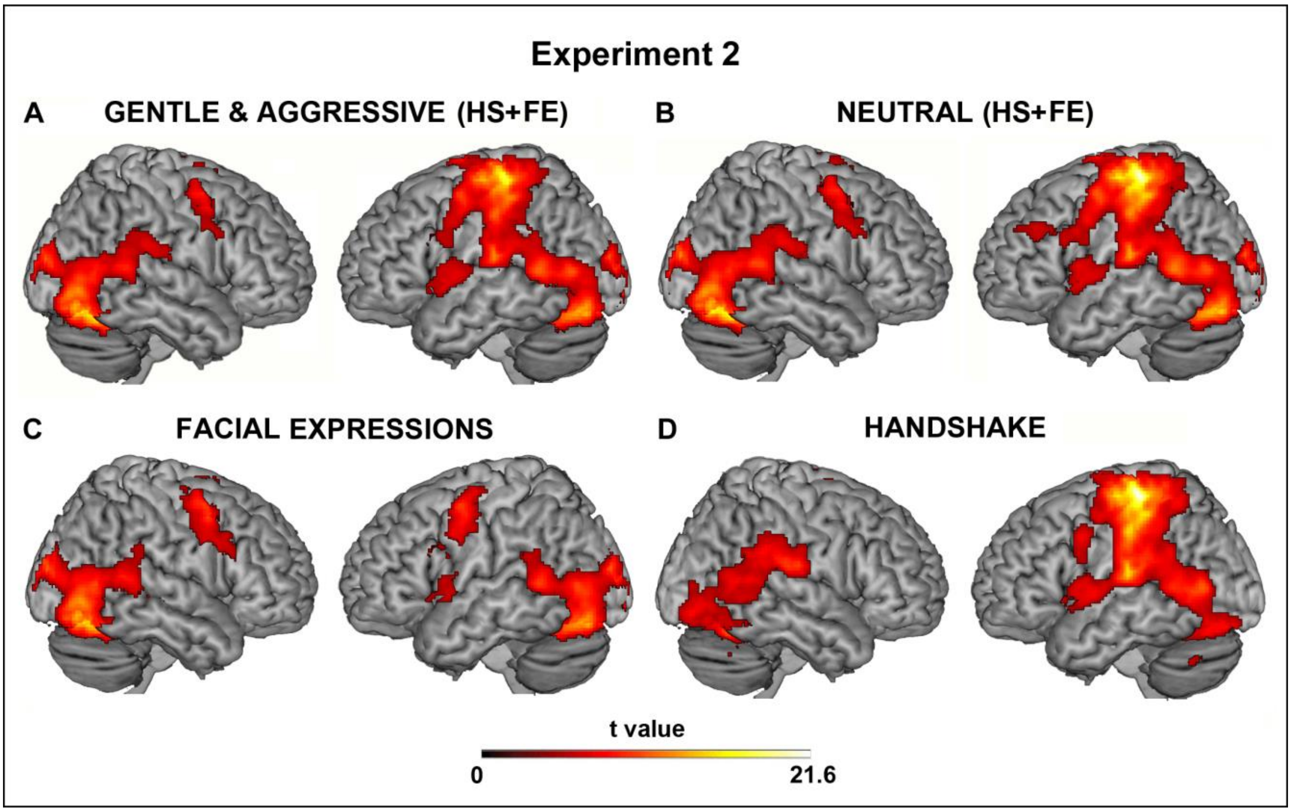

Figure S4. Brain activations obtained in the Exp. 2 from the three different situations (*handshake & facial expression; facial expression; handshake*). Activations obtained in the first situation (HS + FC) during the *vitality forms* (*aggressive* and *gentle*) (A) and neutral (B) (*control*) conditions vs. baseline. Activations obtained in the second (facial expression) (C) and third situations (handshake) (D) vs. baseline. These activations are rendered into a standard Montreal Neurological Institute brain template (P_{FWE} <0.05 voxel level). LH, left hemisphere; RH, right hemisphere.

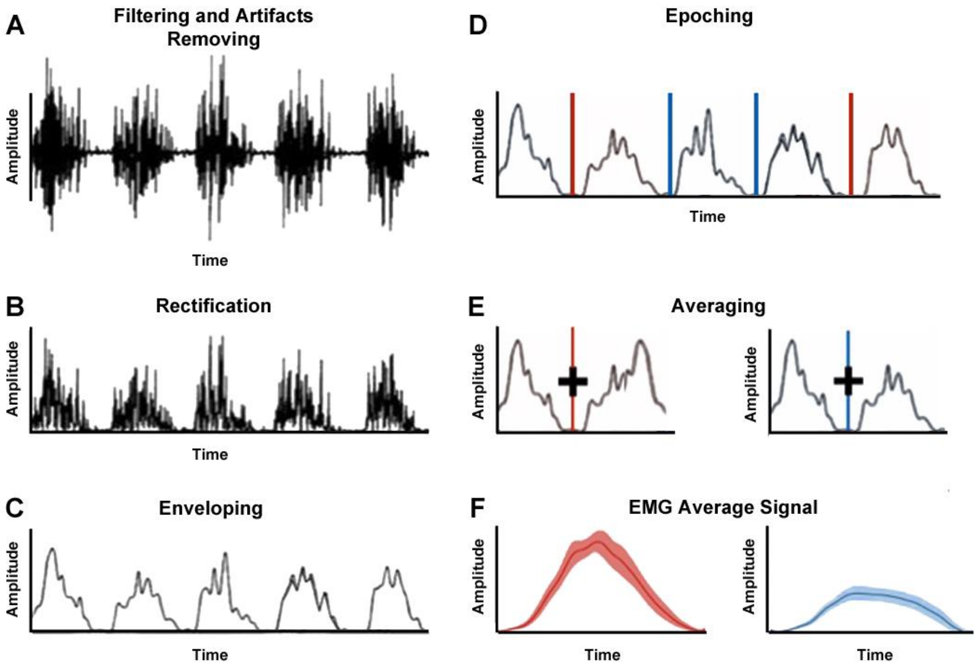

Figure S5. EMG data processing. (A) pass-band filtering and artefacts removal of the raw signal. (B-C) Rectification and root mean square envelope of the filtered signal. (D) Extraction of vigorous, kind and neutral epochs. (E-F) Alignment and averaging of vigorous, kind and neutral EMG stimuli.
